# *Beta vulgaris* L. (Beetroot) Methanolic Extract Prevents Hepatic Steatosis and Liver Damage in T2DM Rats by Hypoglycemic, Insulin-Sensitizing, Antioxidant Effects, and Upregulation of PPARα

**DOI:** 10.3390/biology10121306

**Published:** 2021-12-09

**Authors:** Laila Naif Al-Harbi, Ghedeir M. Alshammari, Alhanouf Mohammed Al-Dossari, Pandurangan Subash-Babu, Manal Abdulaziz Binobead, Maha H. Alhussain, Sahar Abdulaziz AlSedairy, Doha M. Al-Nouri, Ghalia Shamlan

**Affiliations:** Department of Food Science and Nutrition, College of Food Science and Agriculture, King Saud University, Riyadh 11451, Saudi Arabia; aghedeir@ksu.edu.sa (G.M.A.); 438203060@student.ksu.edu.sa (A.M.A.-D.); sbpandurangan@ksu.edu.sa (P.S.-B.); mbinobead@ksu.edu.sa (M.A.B.); mhussien@ksu.edu.sa (M.H.A.); ssudairy@ksu.edu.sa (S.A.A.); dr_nouri@ksu.edu.sa (D.M.A.-N.); Shamlana@ksu.edu.sa (G.S.)

**Keywords:** beetroot, NAFLD, liver, lipid, antioxidant, SREBP1, PPARα

## Abstract

**Simple Summary:**

Beetroot is one of the most consumable plants across the world. Previous studies have shown many health benefits of beetroot, with evidence of having potent hypoglycemic, antioxidant, and anti-inflammatory effects. The data obtained from this study further confirmed this effect in streptozotocin-diabetic animals. They showed the ability of methanolic beetroot extract to prevent the associated hepatic oxidative stress, inflammation, steatosis, and dyslipidaemia. However, the protection mechanisms involve, at least, upregulation of endogenous antioxidants, anti-apoptotic Bcl2, and PPARα.

**Abstract:**

The present study examined if methanolic beetroot extract (BE) could prevent dyslipidemia and hepatic steatosis and damage in a type-2 diabetes mellitus (T2DM) rat model and studied some mechanisms of action. T2DM was induced in adult male Wistar rats by a low single dose of streptozotocin (STZ) (35 mg/kg, i.p) and a high-fat diet (HFD) feeding for 5 weeks. Control or T2DM rats then continued on standard or HFDs for another 12 weeks and were treated with the vehicle or BE (250 or 500 mg/kg). BE, at both doses, significantly improved liver structure and reduced hepatic lipid accumulation in the livers of T2DM rats. They also reduced body weight gain, serum glucose, insulin levels, serum and hepatic levels of cholesterol, triglycerides, free fatty acids, and serum levels of low-density lipoproteins in T2DM rats. In concomitant, they significantly reduced serum levels of aspartate and alanine aminotransferases, hepatic levels of malondialdehyde, tumor-necrosis factor-α, interleukin-6, and mRNA of Bax, cleaved caspase-3, and SREBP1/2. However, both doses of BE significantly increased hepatic levels of total glutathione, superoxide dismutase, and mRNA levels of Bcl2 and PPARα in the livers of both the control and T2DM rats. All of these effects were dose-dependent and more profound with doses of 500 mg/kg. In conclusion, chronic feeding of BE to STZ/HFD-induced T2DM in rats prevents hepatic steatosis and liver damage by its hypoglycemic and insulin-sensitizing effects and its ability to upregulate antioxidants and PPARα.

## 1. Introduction

Type-2 diabetes mellitus is a chronic disorder that results mainly from the ineffectiveness of insulin peripheral action or resistance [1]. T2DM is associated with serval tissue damage and is a leading cause for the development of non-alcoholic fatty liver disease (NAFLD) due to increased hepatic de novo lipogenesis [2]. Yet, NAFLD remains the most prevalent hepatic disease, with an overall global prevalence of more than 37% [3]. However, obesity, due to high-calorie intake and physical inactivity are the major risk factors for developing T2DM and NAFLD [4]. Other common risk factors include genetic and environmental factors, infection, and altered gut microbiota [4].

Dyslipidemia and hepatocyte damage are major (and the earliest) consequences associated with NAFLD [2]. Hepatic oxidative stress and inflammation, mainly due to the co-existence of peripheral insulin resistance (IR), are the key pathological mechanisms leading to hepatic damage in NAFLD patients by acting in a vicious cycle [4,5,6]. Within this view, the influx of free fatty acids (FAs) and inflammatory cytokines/adipokines is highly increased to the liver, causing a state of inflammation, stimulating the generation of reactive oxygen species (ROS) by activating the Kupffer cells, increasing macrophage infiltration, damaging the mitochondria, activating lipogenesis, and promoting endoplasmic reticulum stress [4,7,8,9,10]. ROS and inflammatory cytokines cause hepatic IR, stimulate de novo lipogenesis, and promote hepatocyte damage and fibrosis by impairing insulin signaling and activating several lipogenic, apoptotic, and fibrotic signaling pathways [4,5,6,7,9,11].

Of interest, the process of lipid synthesis in the livers is a tightly regulated mechanism that is controlled by several transcriptional factors. In the liver, the sterol regulator element-binding proteins (SREBPs), including SREBP1c and SREBP2, are a family of transcription factors that stimulates the synthesis of cholesterol (CHOL) and triglycerides (TGs), respectively, in response to insulin, glucose, FFAs, and ROS [12]. However, the peroxisome proliferator-activated receptor-alpha (PPARα) is the major transcriptional factor that stimulates the mitochondria FAs oxidation by activating the mitochondrial carnitine system [13]. In patients and animal models of NAFLD, authors have shown sustained activation of SREBP1 with concomitant downregulation of PPARα, which were shown to be the major hallmarks of the disease [14,15,16]. However, suppressing SREBPs and/or activation of PPARα are effective strategies to prevent NAFLD [14,15,16].

Lifestyle modification and the use of insulin sensitizers, antioxidants, and anti-inflammatory drugs protected against NAFLD and liver damage in experimental animals, showing promising results in human trials [9,10,17,18]. *Beta vulgaris* L. (beetroot) is a common herbal plant found mainly in Europe, Asia, and America. At present, several health benefits of beetroot have been reported in several experimental and clinical studies, including antioxidant, anti-inflammatory, anti-tumorigenesis, anti-diabetic, nephroprotective, hepatoprotective, diuretics, and hypoglycemic potential [19,20,21,22,23,24,25]. Moreover, beetroot has shown potent hepatoprotective and hypolipidemic effects in rats and rabbits fed high cholesterol (CHOL) diets [22,24,26]. Moreover, daily administration of beetroot juice to NAFLD patients significantly reduced serum levels of fasting glucose and lipids and attenuated the increase in some liver transaminases [27]. Similar results, with an improvement in the degree of hepatic steatosis and body weight, were shown in obese rats fed a high-fat diet (HFD) [28].

In rodents, the chronic consumption of HFD that is accompanied by a single early low dose of the diabetic agent, streptozotocin (STZ), is the most common method to induce NAFLD [29]. However, the protective effect of beetroot against NAFLD and hepatic damage in diabetic animals was poorly investigated. In addition, the mechanism underlying the beetroot hypolipidemic effect is still unclear.

Therefore, we investigated the protective effects of beetroot methanolic extract against the development of NAFLD in an HDF/STZ (T2DM) animal model. Moreover, we investigated the possible mechanisms of action by targeting its hepatic effect on markers of oxidative stress and lipogenesis (SREBP1/2 and PPARα).

## 2. Materials and Methods

### 2.1. Animals

All rats used in this study were adult male Wistar rats, aged 10 weeks and weighing 200 ± 20 g. All animals were provided from (and kept in) the animal Department at King Saud University, Riyadh, Saudi Arabia. Housing conditions were standardized at 22 ± 2 °C with a humidity ratio of 50%. During the days of the experiments, the rats had free access to their diets and drinking water. Ethical confirmation was approved by the institutional animal use committee (IRB no.: KSU-SE-21-21).

### 2.2. Animal Diets

All diets used in this study were purchased from Research Diets Inc., New Brunswick, NJ, USA. The standard diet (category number (cat. no.) D12450B) contained 3.82 kcal/g (10% fat), whereas the HFD (cat. no. D12451) contained 4.7 kcal/g (45% fat). The ingredients of both diets are available on the company’s website.

### 2.3. Beetroot Extract Preparation

Freshly cultivated beetroot stems were purchased from a certified local supplier in Riyadh, Saudi Arabia, and the methanolic extract was prepared as described previously [30]. Briefly, all stems were washed with water, the skin was removed, and the pulp was chopped into smaller slices (1 mm). Then, all stem parts were dried at 40 °C and blended to yield a powder soaked in methanol 1:10 (*w*/*v*) for 48 h at 4 °C. The extract was filtered, and the solvent was removed under a vacuum. The extract was then lyophilized and kept at −20 °C until use.

### 2.4. Induction of T2DM

T2DM was induced in rats was conducted as per the protocol by Dwivedi and Jena [31]. In brief, streptozotocin (STZ) (cat. no. sc-200719A, Santa Cruz Biotechnology, Dallas, TX, USA) was freshly dissolved in sodium citrate buffer (0.1 M/pH = 5.5). The animals were fed HFD diets for 2 weeks. By the end of the 2 weeks, the animals were intraperitoneally (i.p.) injected with the prepared STZ solution (35 mg/kg) and continued their HFD for another 3 weeks. By the end of week 5, blood glucose, triglycerides (TGs), and cholesterol (CHOL) levels were measured. Animals with blood glucose higher than 250 mg/dl were considered to have T2DM. Control rats were fed a standard diet for the first 5 weeks and were injected with a single equivalent volume of sodium citrate buffer as the vehicle on the first day of week 2.

### 2.5. Experimental Design

**Figure 1:**
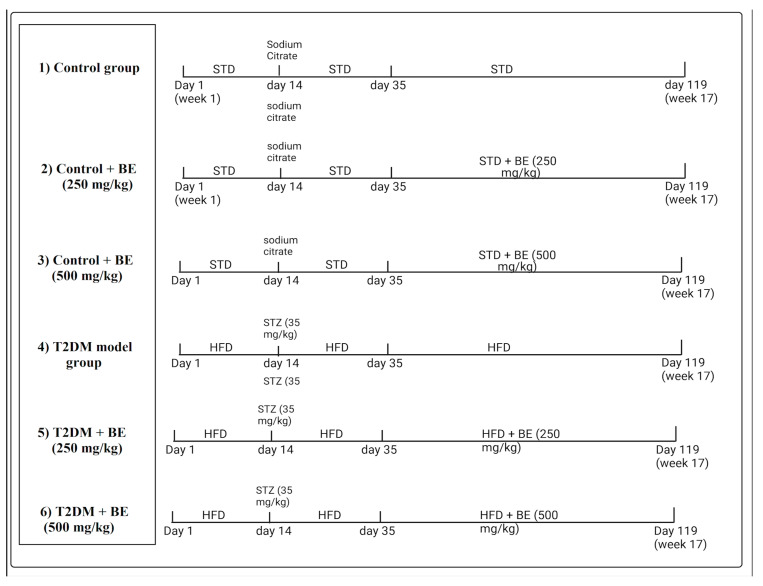
A diagram showing the different experimental groups and experimental design included in this study.

By the end of week 5, the control or T2DM-induced animals continued with their desired diets and were treated for another 12 weeks, as follows, for (1) control rats: fed only standard diets and administered 5% carboxymethyl cellulose (CMC) as a vehicle. (2) Control + BE (250 mg/kg): continued on a standard diet and orally administered beetroot extract (250 mg/kg/day). (3) Control + BE (500 mg/kg): continued normal died and orally given beetroot extract alone (500 mg/kg/day). (4) T2DM model group: continued on HFD and orally administered 5% CMC. (5) T2DM + BE (250 mg): rats with pre-established T2DM that continued HFDs, and were concomitantly orally treated with beetroot extract (250 mg/kg/day). (6) T2DM + BE-(500 mg/kg): rats with pre-established T2DM that continued HFDs, and were concomitantly orally treated with beetroot extract (250 mg/kg/day). Treatment with BE was conducted orally by gavage. Throughout the experiment, the body weight of the rats was recorded every week. Each group contained 8 rats. A presentative diagram of the experimental design and classification of the various groups is shown in Figure 1.

### 2.6. Collection of Blood and Tissue Samples

By the last day of week 17—the rats fasted overnight, and were anesthetized with ketamine/xylazine hydrochloride solution (90:10 mg/kg) [32]. Blood samples (1 mL) were collected from the heart in plain tubes and centrifuged at 3000 rpm (10 min). The serum was collected and stored at −20 °C for further biochemical analysis. The livers were collected on ice and cut into smaller pieces, some of which were kept in 10% buffered formalin, and the remaining were frozen at −80 °C for biochemical and molecular analysis.

### 2.7. Extraction of Lipids from the Liver

Freshly collected livers (*n* = 8/group) were directly used to extract lipid using the methanol: chloroform: normal saline method, as described by Folch et al. [33]. Briefly, liver tissues were homogenized in methanol:chloroform solution (1:2 *v*/*v*) (0.25 g/10 mL) and kept at 4 °C for 1 h. After filtration, normal saline (2 mL) was added to each tube, mixed well, centrifuged (3800 rpm/10 min), and the lower organic layer was isolated. The solvent was evaporated, and the solid material containing the lipids was dissolved in 0.5 mL isopropanol and used for different lipid quantification.

### 2.8. Biochemical Analysis

Serum levels of glucose and insulin were measured using the Rat colorimetric and ELISA kits (cat. no. 10009582 Cayman Chemical, MI, USA, and cat. no. 589501, Ann Arbor, MI, USA, respectively). The homeostasis model of insulin resistance was calculated as described by Salgado et al. [34] using the following formula—HOMA-IR fasting insulin (ng/mL) × fasting glucose (mg/dl)/405. Hepatic tissue was homogenized in ice-cold PBS (pH = 7.4) and centrifuged at a speed of 11,500 rpm at 4 °C for 15 min; the supernatant containing tissue extract was collected and used to measure biochemical parameters. Serum and hepatic CHO, TGs, high-density lipoprotein cholesterol (HDL-c), and low-density lipoprotein cholesterol (LDL-c) levels were determined using Rat specific assay kits (cat. no. ECCH-100, BioAssay Systems, CA, USA, cat. no. 10010303, Cayman Chemical, MI, USA, cat. no. K4436, BioVision, CA, USA, and cat. no. 79960, Crystal Chemicals, PA, USA, respectively). Serum alanine aminotransferase (ALT), aspartate aminotransferase (AST) activities, hepatic malondialdehyde (MDA), glutathione (GSH), and superoxide dismutase (SOD) levels were measured using Rat ELISA kits (cat. no. MBS269614; cat. no. MBS264975, cat. no. MBS268427, cat. no. MBS738685, cat. no. MBS265966, MyBioSource, CA, USA, respectively). Hepatic levels of tumor necrosis factor-alpha (TNF-α) and interleukin 6 (IL-6) were measured using Rat ELISA kits (cat. no. MBS2507393; cat. no. MBS175908, MyBioSource, CA, USA, respectively).

### 2.9. Real-Time PCR

Real-time PCR measured the mRNA expression of some lipogenic and apoptotic factors (Table 1). All primers were purchased from (Promega, WI, USA). Primer sequences related genes are provided in Table 1. Total RNA has extracted the 0.25 mg frozen livers using ReliaPrep™ RNA Miniprep Systems (cat. no. Z6010, Promega, WI, USA). The template cDNA of each sample was synthesized with the help of GoScript synthesis kit (cat. no. A5001, Promega, WI, USA). Amplification was conducted in a real-time PCR machine (model: CFX, Bio-Rad, CA, USA) using the Ssofast EvaGreen Supermix kit and instrument setting provided in a 96-well plate. All procedures were conducted as per each kit’s instructions. Two samples with no template cDNA were included as negative controls. The amplification of each target was normalized to its corresponding mRNA levels of β-actin using the −2ΔΔCT method on the available software.

### 2.10. Histopathological Analysis

Alterations in liver morphology were determined by staining. Formalin-preserved tissues were hydrated and cleared with ethanol and xylene. Then, they were embedded in paraffin, cut at 3–5 µM, and routinely stained with hematoxylin and eosin (H&E). All photos were captured under a light microscope.

### 2.11. Statistical Analysis

GraphPad Prism statistical software (V8, Australia) was used for statistical analyses. Normality was tested using the Shapiro–Wilk test. All analyses were conducted using a two-way analysis of variance (ANOVA) followed by Tukey’s post hoc test. The values were presented as mean ± standard deviation (SD) and were considered significantly different at *p* < 0. 05.

## 3. Results

### 3.1. BE Ameliorates the Gain in Body Weight and Reduces Fasting Glucose and Insulin Levels in T2DM Rats

As shown in Table 2, the final body weights, fasting blood glucose levels, fasting insulin levels, and HOMA-IR were not significantly different between the control and control + BE (250 mg/kg). However, only fasting blood glucose levels and HOMA-IR were significantly lower in control + BE (500 mg/kg)-treated rats compared to control rats (Table 2). A significant increase in the final body weights, fasting glucose and insulin levels, and values of HOMA-IR were seen in T2DM-induced rats when compared to control rats, which were then significantly reduced in T2DM, and treated with BE at doses of 250 or 500 mg/kg (Table 2). However, the reduction in all of these parameters was more significant in T2DM + BE (500 mg/kg)-treated rats when compared to T2DM + BE (250 mg/kg)-treated rats, levels that were not significantly different when compared to control rats (Table 2).

### 3.2. BE Attenuates T2DM-Induced Dyslipidemia

Serum and hepatic levels of FFAs, TGs, and CHOL, as well as serum levels of LDL-c, were significantly higher in T2DM-induced rats when compared to control rats (Table 3). However, serum and hepatic levels of FFAs, TGs, and CHOL, and levels of LDL-c were significantly lower in both control + BE (250 and 500 mg/kg) and T2DM + BE (250 and 500 mg/kg)-treated rats when compared to either the control or T2DM-induced rats (Table 3). Levels of all these biochemical markers were significantly lower in control + BE (500 mg/kg) and T2DM + BE (500 mg/kg) when compared to the control or T2DM rats, which received the lower dose of the extract (250 mg/kg) (Table 3).

### 3.3. BE Attenuates Oxidative Stress in the Livers of T2DM Rats

**Figure 2:**
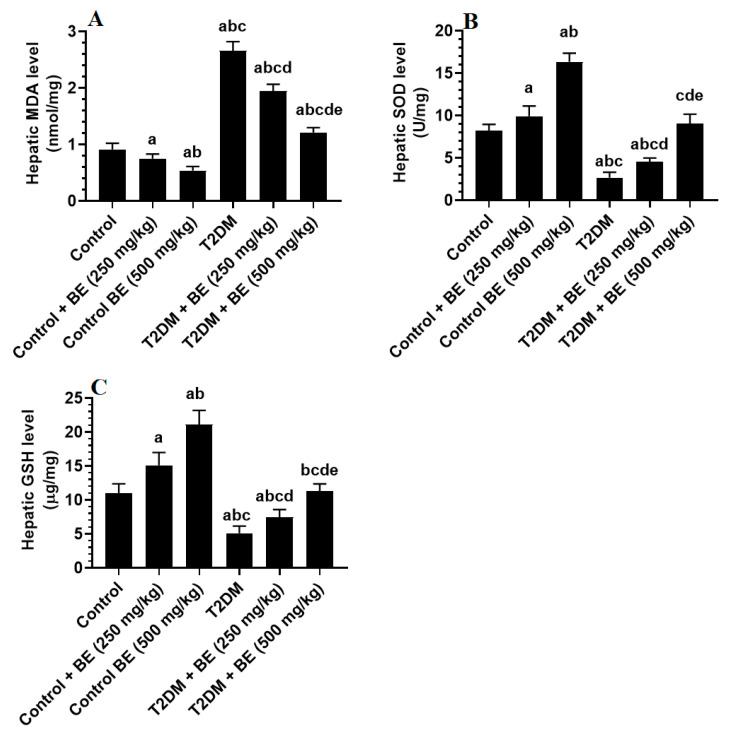
Levels of malondialdehyde (MDA) (**A**), superoxide dismutase (SOD) (**B**), and total reduced glutathione (GSH) (**C**) in the livers of all groups of rats. Data were analyzed by one-way ANOVA followed by Tukey’s test as post hoc and presented as mean ± SD of eight rats/group. ^a^: vs. the control rats. ^b^: vs. the control + beetroot extract (BE) (250 mg/kg)-treated rats. ^c^: vs. the control + BE (500 mg/kg)-treated rats. ^d^: vs. T2DM-treated rats. ^e^: vs. T2DM + BE (250 mg/kg).

Levels of MDA were significantly higher, but levels of SOD and GSH were significantly lower in the livers of T2DM-induced rats compared to control rats (Figure 2A–C). However, levels of MDA were significantly lower, and levels of SOD and GSH were significantly higher in the livers of both the control and T2DM rats, which received either the lower or higher doses of the BE (250 and 500 mg/kg) when compared to either the control or T2DM-induced rats, respectively (Figure 2A–C). In addition, the reduction in levels of MDA and the increase in the levels of SOD and GSH were significantly higher in control + BE (500 mg/kg) and T2DM + BE (500 mg/kg) when compared to control + BE (250 mg/kg) and T2DM + BE (350 mg/kg), respectively (Figure 2A–C). While levels of MDA remained slightly but significantly reduced, levels of SOD and GSH were not significantly different between T2DM + BE (500 mg/kg)-treated rats and control rats (Figure 2A–C).

### 3.4. BE Attenuates Serum Transaminases and Hepatic Inflammatory Markers in T2DM Rats

**Figure 3:**
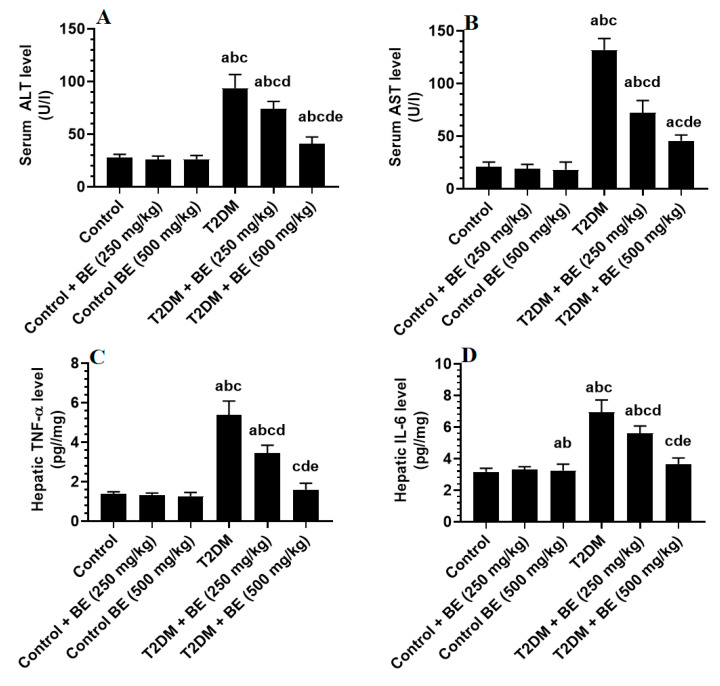
Serum levels of alanine aminotransferase (ALT) (**A**), aspartate aminotransferase (AST) (**B**), hepatic levels of tissue necrosis factor dismutase (TNF-α) (**C**), and interleukin-6 (IL-6) (**D**) in all groups of rats. Data were analyzed by one-way ANOVA followed by Tukey’s test as post hoc and presented as mean ± SD of eight rats/group. ^a^: vs. the control rats. ^b^: vs. the control + beetroot extract (BE) (250 mg/kg)-treated rats. ^c^: vs. the control + BE (500 mg/kg)-treated rats. ^d^: vs. T2DM-treated rats. ^e^: vs. T2DM + BE (250 mg/kg).

Serum levels of ALT and AST and hepatic levels of TNF-α and IL-6 were not significantly different between control and control + BE (250 and 500 mg/kg) (Figure 3A–D). Levels of ALT and AST and hepatic levels of TNF-α and IL-6 were significantly higher in T2DM-induced rats compared to control rats, but progressively decreased in T2DM + BE (250 and 500 mg/kg) when compared to T2DM-induced rats (Figure 3A–D). Serum levels of ALT remained slightly increased, whereas serum levels of AST and hepatic levels TNF-α and IL-6 were not significantly different when T2DM + BE (250 mg/kg)-treated rats were compared to control rats (Figure 3A–D).

### 3.5. BE Downregulates the Transcription SREBP1/2, Stimulates PPARα, and Inhibits Intrinsic Cell Apoptosis in the Livers of T2DM Rats

**Figure 4:**
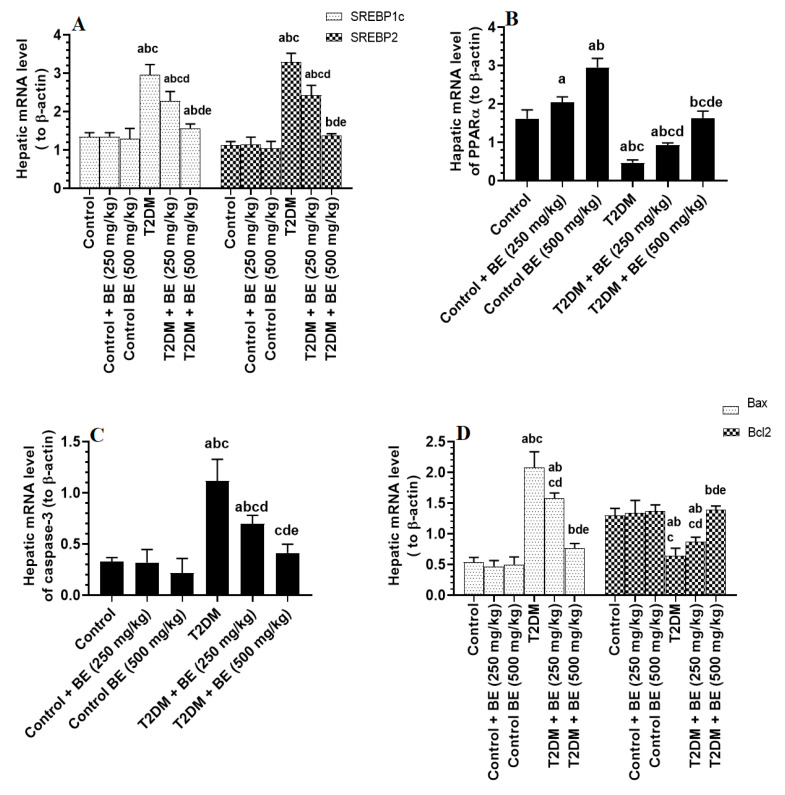
mRNA levels of SREBP1 (**A**), SREBP2 (**A**), PPARα (**B**), caspase-3 (**C**), and Bax (**D**), Bcl2 (**D**) in the livers of all groups of rats. Data were analyzed by one-way ANOVA followed by Tukey’s test as post hoc and presented as mean ± SD of eight rats/group. . ^a^: vs. the control rats. ^b^: vs. the control + beetroot extract (BE) (250 mg/kg)-treated rats. ^c^: vs. the control + BE (500 mg/kg)-treated rats. ^d^: vs. T2DM-treated rats. ^e^: vs. T2DM + BE (250 mg/kg).

Hepatic mRNA levels of SREBP1c, SREBP2, Bax, Bcl2, and caspase-3 did not significantly change between the control and control + BE (250 or 500 mg/kg) (Figure 4A,C,D). The mRNA levels of PPARα were significantly higher in the livers of both control + BE (250 mg/kg) and control + BE (500 mg/kg) when compared to control rats, with a higher significant increase seen with the higher dose of the extract (Figure 4A–D). mRNA levels of SREBP1, SREBP2, Bax, Bcl2, and caspase-3 were significantly higher, but mRNA levels of PPARα were significantly lower in the livers of T2DM-induced rats compared to control rats (Figure 4A–D). The mRNA levels of SREBP1, SREBP2, Bax, Bcl2, and caspase-3 were significantly lower, but mRNA levels of PPARα were significantly higher in the livers of both T2DM + BE (250 mg/kg) and T2DM (500 mg/kg)-treated rats when compared to T2DM rats (Figure 4A–D). The improvement in all these markers was significantly higher in T2DM + 500 mg/kg-treated rats when compared to those that received the lower dose of the extract (250 mg/kg), levels that were not significantly different when compared to control rats (Figure 4A–D).

### 3.6. BE Improved Liver Architectures of T2DM Rats

**Figure 5:**
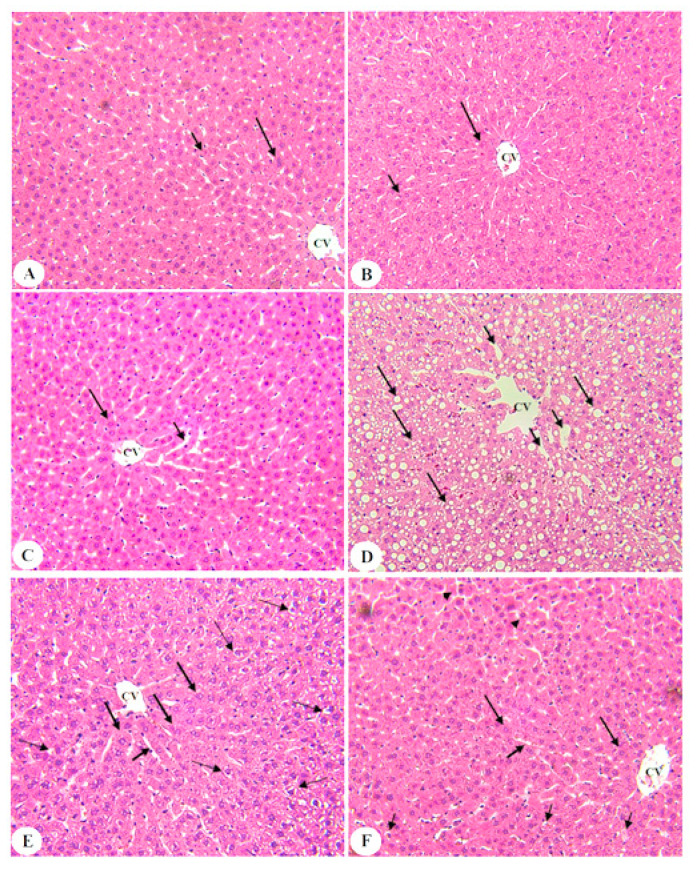
Photomicrographs of the liver section of all groups of rats as stained by hematoxylin and eosin (H&E) staining; 200 ×. (**A**–**C**) show the control, control + BE (250 mg/kg), and control + BE (500 mg)-treated rats and showing normal hepatocytes structure (long arrow) radiating from a central vein (CV). Note the normally sized sinusoids (short arrow). (**D**): was taken from a T2DM-induced rat and showed dilated CV with increased cytoplasmic fat accumulation and ballooning (long arrow) in the hepatocytes. The sinusoids were abnormally dilated (short arrow). (**E**): was taken from a T2DM + BE (250 mg/kg)-treated rats and showed an improvement in the structure of the hepatocytes. Although some livers appear normal with no fat accumulation (long thick arrow), many hepatocytes remained filled with fat droplets (long thin arrow). In addition, most of the sinusoids appeared normally sized (short arrow). (**F**): was taken from a T2DM + BE (500 mg/kg) and showed almost normal morphology with those observed in control, with normal hepatocytes (long arrow) and sinusoids (short arrow).

Normal histological architectures with normal hepatocytes, central vein (CV), and sinusoids were seen in the control, control + BE (250 mg/kg), and control + BE (500 mg/kg)-treated rats (Figure 5A–C, respectively). Livers of T2DM induced rats showed severe accumulation of fat droplets in the cytoplasm of the hepatocytes with dilated CV and sinusoids (Figure 5D). An improvement in the structure of the livers with a significant reduction in lipid accumulation was seen in the livers of T2DM + BE (250 mg/kg)-treated rats (Figure 5E). However, almost normal morphology of the liver section was seen in the livers of T2DM + BE (500 mg/kg)-treated rats (Figure 5F).

## 4. Discussion

T2DM is characterized by a significant increase in final body weights, sustained hyperglycemia, hyperinsulinemia, and IR and is associated with NAFLD [35]. In rodents, STZ-induced DM is the most acceptable model to study the complication of T2DM and NAFLD [36,37]. Using this animal model, simple steatosis is seen after 6–8 weeks, whereas NASH characterized by hepatocellular ballooning is seen after 8–12 weeks [29]. At this stage, the animals also show an increase in their final body weight, fasting hyperglycemia, hyperinsulinemia, dyslipidemia, and increased transaminase levels [29,38]. These clinical manifestations were also observed in the STZ/HFD treated rats of this study, thus validating our animal model. However, the attenuation of all these markers in beetroot-treated T2DM rats was our solid and initial evidence for this plant’s anti-diabetic and hepatoprotective effects. Moreover, our data showed that this protection occurs in a dose-dependent manner where the higher dose of the extract (500 mg/kg) showed more profound results.

Insulin is the major metabolic hormone that regulates blood glucose levels by inhibiting gluconeogenesis, stimulating glycogen synthesis, peripheral glucose disposal, and adipogenesis [39]. Under IR, as in HFD-induced obesity, hyperglycemia dominates, and the delivery of FFAs and inflammatory cytokines from the periphery to the liver is increased, thus stimulating the generation of ROS and inflammatory cytokines by activating numerous pathways [4,6]. These ROS and inflammatory cytokines act viciously to induce hepatic IR and hepatocytes apoptosis [4]. In addition, glucose is continuously produced by the liver due to oxidative/inflammation-induced impairment in insulin signaling [40]. In the same line, the observed increment in the levels of ROS, MDA, TNF-α, and IL-6, and the parallel reduction in the levels of the antioxidants (i.e., GSH and SOD) in the livers of T2DM-induced rats support the indispensable roles of oxidative stress and inflammation in mediating liver damage under HFD, and diabetic conditions, which support others [41,42,43,44,45]. In addition, T2DM-induced rats showed a significant increase in the mRNA levels of markers of intrinsic cell apoptosis (Bax and caspase-3) with a concomitant reduction in the levels of Bcl2, which indicates the activation of intrinsic cell death. Indeed, several lines of evidence have shown that mitochondria-mediated cell apoptosis is the most common cell modality in the livers of animal models of NAFLD, and is mediated by oxidative stress and inflammation [46,47,48].

The treatment with the highest dose of beetroot extract reversed the alterations in these oxidants, inflammatory, and apoptotic markers, and concomitantly reduced fasting circulatory glucose and insulin levels, as well as the hepatic and serum FFAs levels. Such reduction in the levels of FFAs with the concomitant decrease in HOAM-IR values in the T2DM-treated rats is a clear indication for the improvement of peripheral insulin sensitivity. However, although the lower dose failed to alter insulin levels, the levels of TNF-α or IL-6, apoptotic markers in the control, or T2DM-treated rats—it reduced fasting glucose levels in control rats, thus suggesting a potent hypoglycemic effect. These data may indicate the ability of the beetroot to reduce either glucose absorption or hepatic synthesis. Moreover, both doses of the beetroot extract stimulated levels of SOD and GSH in the livers of control rats, suggesting an independent antioxidant effect that may mediate its anti-inflammatory effect. Therefore, we could strongly argue that the hepatoprotective effect of beetroot extract in STZ/HFD-induced T2DM rats is mediated by a concomitant hypoglycemic, insulin sensitizing, and antioxidant potential.

Supporting our data, several lines of evidence have also shown the potent hypoglycemic, anti-inflammatory, and antioxidant potentials of beetroot juice or extracts in a variety of animal models [49,50]. Indeed, daily consumption of red beetroot juice extracts significantly reduced fasting glucose levels in diabetic individuals, as well as in STZ and alloxan-induced diabetic animals, effects that were attributed to increased insulin release (in T1DM), decreased glucose absorptions, stimulated insulin sensitivity, GLUT-2/4 receptor expression, and inhibited hepatic glucose synthesis [25,30,51,52,53]. Moreover, the antioxidant protective effects of beetroots/juice extracts were reported in the livers of several animal models of liver injury, including those induced by chlorpyrifos (CPF), carbon tetrachloride (CCl4), 7,12-dimethylbenz[a]anthracene (DMBA), N-nitrosodiethylamine (NDEA), and were shown to be mediated by direct scavenging and inhibiting or ROS, suppressing lipid peroxidation upregulation of GSH, and phase II antioxidant enzymes (e.g., glutathione peroxidase (GPx), catalase, glutathione reductase (GSHR), glutathione transferase) [54,55,56,57,58,59]. However, the stimulatory effect of beetroot on the levels of GSH and other antioxidant enzymes was attributed to upregulation of the nuclear factor-erythroid factor 2-related factor 2 (Nrf2), a major antioxidant transcription factor [30,50,60]. In addition, beetroot extract also reduced the generation of RSOS, TNF-α, and IL-6, inhibited NF-κB, enhanced catalase and Bcl2, and reduced the levels of Bax and cleaved caspase-3 in the kidneys of gentamicin-treated rats [61]. It significantly reduced the hepatic levels of inflammatory markers and reduced circulatory levels of AST, ALT, lactate dehydrogenase (LDH), and gamma-glutamyl transferase (γGTT) in the livers of NDEA-treated rats [59,62]. Moreover, extracts of beetroots significantly reduced levels of AST in patients with NAFLD [27].

In the liver, the regulation of SREBP1/2 is regulated by insulin [63]. SREBP1c and SREBP2 stimulate de novo lipid synthesis by activating several lipogenic genes in the liver. Indeed, SREBP1c stimulates FAs and CHOL synthesis by upregulating FAS, and ACC-1 whereas SREBP2 stimulates CHOL synthesis by activating the HMGCR [12]. PPARα is an anti-lipogenic transcription factor that stimulates the mitochondria FAs uptake and oxidation by activating the L-carnitine system [13]. Fasting hyperglycemia and increased influx of FFAs, as well as higher levels of ROS and inflammatory cytokines in the liver, stimulate SREBP1/2 in the livers of NAFLD patients and animals, even independent of insulin resistance [64,65,66,67].

The increased FA oxidation suppresses PPARα in patients or animal models of NAFLD [68]. In the same line as these studies, levels of SREBP1/2 were significantly lower, whereas levels of PPARα were significantly higher in the livers of STZ/HFD-treated rats, which explains the concomitant increase in hepatic fat droplet accumulation and the higher levels of CHOL and TGs in the livers and serum of rats. Such activation of SREBP1c/2 could be explained by the development of hepatic IR, as well as by the effects afforded by hyperglycemia, ROS, and inflammatory cytokines. However, the significant reduction in the levels of PPARα in the livers of these diabetic rats could be attributed to the increase in hepatic levels of FFAs, which probably increased peripheral influx, or due to the activation of SREBP1c.

The hypolipidemic effect of beetroot juice or extract is well-reported in healthy individuals, rodents, rabbits fed a “cholesterol diet”, and diabetic animals [51,52,69,70,71,72]. In most of these studies, the beneficial effects of beer root included lowering circulatory CHOL, TGs, and LDL-c levels and increasing HDL-c levels. Similar supporting results were also demonstrated in the livers of the control and T2DM-induced rats of this study, which were treated with both doses of the extract. Moreover, at both low and higher doses, beetroot extract also reduced hepatic FFAs, CHOL, and TGs in the serum and livers of the control and T2DM-treated rats. Yet, the mechanisms of such effect are poorly investigated and remain unknown. However, and as discussed previously, the significant reduction in the levels of FFAs could be explained by the improvement of peripheral insulin sensitivity and the subsequent decrease in the adipose tissue lipolysis.

The novelist finding of this study is the ability of both doses of the beetroot extract to attenuate the increase in the levels of SREBP1/2 and stimulate PPARα to attenuate dyslipidemia hepatic lipid synthesis in T2DM-induced rats. These effects could be explained by the hypoglycemic, insulin-sensitizing, antioxidant, and anti-inflammatory effects of the extract. However, one interesting observation is that both doses of the extract also increased the transcription of PPARα in the livers of control rats. This could explain why the livers and serum of these rats also showed lower CHOL, FFAs, and TGs levels. Hence, the beetroot extract seems more likely to have a direct independent effect on PPARα, which underlies its hypolipidemic effect. As beetroot extract did not affect levels of SREBP1/SREBP2 in the livers of control rats, these findings suggest that the beetroot-induced suppression of these transcription factors occurred indirectly due to its hyperglycemic effect and its ability to attenuate the hepatic oxidative stress, inflammation, and IR in T2DM-rats.

One limitation in this study is that our data were based only on studying the effects of the extract on mRNA levels of the target lipogenic transcription factors. Studying the protein expression of these targets may provide the readers and us with more accurate data for the effect of BE, which should be considered in future studies. Moreover, we were unable to determine the exact constituent of the extract responsible for these effects. Beetroots are rich in compounds that can exert such effects. These include betalains, flavonoids (e.g., gallic acid, betavulgaroside, myricetin, apigenin, luteolin, quercetin, and kaempferol), fibers, and pectin. These ingredients are known for their hypoglycemic, hypolipidemic, and antioxidant potential, and they can modulate numerous cells signaling with the cells they constitute; it is well established in the literature. This has been discussed in previous studies and reviews [49,50,60]. Moreover, further investigation is required to examine the precise molecular mechanisms responsible for the hypoglycemic, hypolipidemic, and antioxidant effects of the extract. One important common target could be the AMPK/SIRT1 axis, which represents the most important energy sensors that regulate glucose and lipid metabolism, as well as the cellular redox potential in most cells [73].

## 5. Conclusions

The findings of this study show that beetroot is an effective therapy to treat NAFLD in rodents. Since NAFLD is a complex disorder with several underlying mechanisms and co-morbidities, the data are encouraging, and may lead to future subclinical and clinical studies, for researchers to examine the effects of the methanolic extract of beetroot in diabetic and metabolically active patients. In addition, these data are encouraging, for researchers to further isolate and identify an active ingredient that could be provided commercially to patients to reduce the socioeconomic burden of this disease, and prevent all associated comorbidities.

## Figures and Tables

**Table 1:** Primer’s characteristics of the real-time PCR.

Gene	Primers (5′→3′)	GenBank Accession #	Product Length
PPARα	F: TGCGGACTACCAGTACTTAGGG R: GCTGGAGAGAGGGTGTCTGT	NM_013196.1	116
SREBP-1c	F: GCA AGG CCA TCG ACT ACA TC R: TTT CAT GCC CTC CAT AGA CAC	NM_001276707.1	161
Bcl2	F: TGGGATGCCTTTGTGGAACT R: TCTTCAGAGACTGCCAGGAGAAA	U34964.1	73
SREBP-2	F: CTGACCACAATGCCGGTAAT R: CTTGTGCATCTTGGCATCTG	NM_001033694.1	204
Bax	F: ATGGAGCTGCAGAGGATGATT R: TGAAGTTGCCATCAGCAAACA	NM_017059	97
Caspase-3	F: AATTCAAGGGACGGGTCATG R: R-GCTTGTGCGCGTACAGTTTC	U49930	67
Β-actin	F: ATC TGG CAC CAC ACC TTC R: AGC CAG GTC CAG ACG CA	NM_031144	291

**Table 2:** Average final body weights (A), levels of fasting glucose (B) and insulin (C), and values of HOMA-IR (D) in all groups of rats.

Items	Control	Control + BE (250 mg/kg)	Control + BE (500 mg/kg)	T2DM	T2DM + BE (250 mg/kg) + CC	T2DM + BE (500 mg/kg)
Final body weight (g)	442 ± 24.3	446 ± 17.5	454 ± 27.6	576 ± 25.4 ^abc^	536 ± 21.6 ^abcd^	451 ± 18.9 ^de^
Plasma fasting glucose (mg/dL)	100 ± 8.6	96 ± 7.8	65 ± 11.3 ^ab^	253 ± 18.5 ^abc^	189 ± 15.1 ^abcd^	121 ± 10.1 ^abcde^
Plasma fasting insulin	4.8 ± 0.71	4.6 ± 0.73	4.7 ± 61	9.3 ± 0.81 ^abc^	6.9 ± 0.41 ^abcd^	5.0 ± 0.56 ^de^
(ng/mL)						
HOMA-IR	1.19 ± 0.23	1.11 ± 0.19	0.75 ± 0.11 ^ab^	5.8 ± 0.82 ^abc^	3.2 ± 0.33 ^abcd^	1.5 ± 0.24 ^abcde^

Data were analyzed by one-way ANOVA followed by Tukey’s test as post hoc and presented as mean ± SD of eight rats/group. ^a^: vs. the control rats. ^b^: vs. the control + beetroot extract (BE) (250 mg/kg)-treated rats. ^c^: vs. the control + BE (500 mg/kg)-treated rats. ^d^: vs. T2DM-treated rats. ^e^: vs. T2DM + BE (250 mg/kg).

**Table 3:** Lipid profiles in the serum and livers of all groups of rats.

	Control	Control + BE (250 mg/kg)	Control + BE (500 mg/kg)	T2DM	T2DM + BE (250 mg/kg) + CC	T2DM + BE (500 mg/kg)
**Serum**						
TGs (mg/dl)	51.1 ± 4.8	40.3 ± 2.8 ^a^	30.5 ± 2.9 ^ab^	120 ± 8.3 ^abc^	85.4 ± 4.2 ^abcd^	55.8 ± 3.6 ^bcde^
CHOL (mg/dl)	79.8 ± 6.1	68.8± 3.9 ^a^	55.1 ± 4.3 ^ab^	162 ± 10.9 ^abc^	136 ± 10.1 ^abcd^	95.2 ± 5.5 ^abcde^
LDL-c (mg/dl)	42.4 ± 4.1	32.1 ± 5.4 ^a^	27.8 ± 4.2 ^ab^	96.1 ± 5.6 ^abc^	77.1 ± 4.6 ^abcd^	54.6 ± 3.7 ^abcde^
FFAs (µM/l)	199 ± 13.1	167 ± 10.3 ^a^	135 ± 7.5 ^ab^	756 ± 114 ^abc^	462 ± 79 ^abcd^	244 ± 32 ^abcde^
**Liver**						
TGs (mg/g)	0.41 ± 0.06	0.33 ± 0.04 ^a^	0.24 ± 0.03 ^ab^	0.88 ± 0.12 ^abc^	0.7 ± 0.06 ^abcd^	0.43 ± 0.05 ^bcde^
CHOL (mg/g)	2.8 ± 0.22	2.11 ± 0.17 ^a^	1.7 ± 0.09 ^ab^	6.3 ± 0.51 ^abc^	4.9 ± 0.36 ^abcd^	3.0 ± 0.26 ^bcde^
FFAs (µM/g)	59.6 ± 7.9	49.1 ± 4.2 ^a^	32.9 ± 3.1 ^ab^	340 ± 27.1 ^abc^	236± 26 ^abcd^	110 ± 16.2 ^abcde^

Data were analyzed by one-way ANOVA followed by Tukey’s test as post hoc and presented as mean ± SD of 8 rats/group. ^a^: vs. the control rats. ^b^: vs. the control + beetroot extract (BE) (250 mg/kg)-treated rats. ^c^: vs. the control + BE (500 mg/kg)-treated rats. ^d^: vs. T2DM-treated rats. ^e^: vs. T2DM + BE (250 mg/kg).

## Data Availability

All data included in this study are available from the corresponding author upon reasonable request.
